# Hospitalizations after the Gulf War—Reply to K.M. Leisure et al.

**DOI:** 10.3201/eid0404.980435

**Published:** 1998

**Authors:** James D. Knoke, Gregory C. Gray

**Affiliations:** Naval Health Research Center, Sand Diego, California, USA


2. Knoke JD, Gray GC. Hospitalizations for unexplained illnesses among U.S. veterans of the Persian Gulf War. Emerg Infect Dis 1998;4:211-19.
3. Haley RW. Bias from the "healthy warrior effect" and unequal follow-up in three government studies of health effects of the Gulf War. Am J Epidemiol 1998;148:315-23.
4. Murray-Leisure KA, Sees J, Zangwill B, Suguitan E, Legaspi C, Bagheri S, Mucha P, Brinser E, Kimber R, Kurban R, Greene W. Mucocutaneous-intestinal-rheumatic Desert Syndrome (MIRDS): Incubation & histopathology findings. Abstract K 48, American Society for Microbiology, ICAAC, Sept. 18, 1995.
5. U.S. General Accounting Office. Gulf War Illnesses: Improved Monitoring of Clinical Progress and Reexamination of Research Emphasis are Needed. GAO/NSIAD-97-163. June 1997.
6. Sostek MB, Jackson S, Linevsky JK, Schimmel EM, Fincke BG. High prevalence of chronic gastrointestinal syndromes in a National Guard unit of Persian Gulf Veterans. Am J Gastroenterol 1996;91:2494-7.
7. Centers for Disease Control. Unexplained illness among Persian Gulf War veterans in an Air National Guard unit: Preliminary report, Aug 1990-March 1995. Morb Mortal Week Rep 1995;44:443-7
8. Fukada K, Nisenbaum R, Stewart G, Thompson WW, Robin L, Washko R, et al. Chronic multisystem illnesses affecting Air Force veterans of the Gulf War. JAMA 1998;280:981-8.
9. Nicolson G, Nicolson N. Gulf War illnesses. Medicine, Conflict, and Survival 1998;14:156-65.
10. Magill AJ, Grogl M, Gasser RA, Wellington S, Oster CN. Viscerotropic leishmaniasis caused by *Leishmania tropica* in soldiers returning from Operation Desert Storm. N Engl J Med 1993;328:1383-7.
11. Magill AJ, Grogl M, Johnson SC, Gasser RA. Visceral infection due to *Leishmania tropica* in a veteran of Operation Desert Storm who presented two years after leaving Saudi Arabia. Clin Infect Dis 1994;19:805-6.
12. Ferrante MA, Dolan MJ. Q Fever meningoencephalitis in a soldier returning from the Persian Gulf War. Clin Infect Dis 1993;16:489-96.
13. Seaman J, Mercer AJ, Sondorp E. Epidemic of visceral Leishmaniasis in western upper Nile, southern Sudan: Course and impact from 1984 to 1994. Int J Epidemiol 1996;25:862-71.
14. Zilinskas RA. Iraq's biological weapons. JAMA 1997;278:418-24.

### Hospitalizations after the Gulf War—Reply to K.M. Leisure et al.

**To the Editor:** We studied all active-duty Persian Gulf War–era veterans who remained on active duty at the conclusion of deployment (July 31, 1991), not as Leisure et al. stated in their letter "selected, mostly healthy, active-duty Persian Gulf War veterans enlisted as of 1994."

Our study was restricted to hospitalizations of active-duty service members because these were the only service members whose records were available on computerized files. No one was excluded from the defined target population. However, there are "sick Gulf War veterans" and healthy Gulf War veterans not in the target population. The difficulty is in studying either a random sample or the entire population of Gulf War veterans. The only published study we know of the entire population is the mortality report of Kang and Bullman (1).

The suggestion that we should have excluded from the control group service members who had ever been in the Gulf War area would have been appropriate for a report of exposure to the Persian Gulf region; ours was a report of exposure to the Persian Gulf War. That we should have studied a different collection of ICD-9 diagnoses also suggests a different report.

While our study may have limitations, we have not seen objective data that support the anecdotal observations of Leisure et al.

**James D. Knoke and Gregory C. Gray**
Naval Health Research Center, Sand Diego, California, USA

### Reference

1. Kang HK, Bullman TA. Mortality among U.S. veterans of the Persian Gulf War. N Engl J Med 1996;335:1498-504.

**To the Editor:** We studied all active-duty Persian Gulf War–era veterans who remained on active duty at the conclusion of deployment (July 31, 1991), not as Leisure et al. stated in their letter "selected, mostly healthy, active-duty Persian Gulf War veterans enlisted as of 1994."

Our study was restricted to hospitalizations of active-duty service members because these were the only service members whose records were available on computerized files. No one was excluded from the defined target population. However, there are "sick Gulf War veterans" and healthy Gulf War veterans not in the target population. The difficulty is in studying either a random sample or the entire population of Gulf War veterans. The only published study we know of the entire population is the mortality report of Kang and Bullman (1).

The suggestion that we should have excluded from the control group service members who had ever been in the Gulf War area would have been appropriate for a report of exposure to the Persian Gulf region; ours was a report of exposure to the Persian Gulf War. That we should have studied a different collection of ICD-9 diagnoses also suggests a different report.

While our study may have limitations, we have not seen objective data that support the anecdotal observations of Leisure et al.
